# Structuring successful collaboration: a longitudinal social network analysis of a translational research network

**DOI:** 10.1186/s13012-016-0381-y

**Published:** 2016-02-11

**Authors:** Janet C. Long, Peter Hibbert, Jeffrey Braithwaite

**Affiliations:** Australian Institute of Health Innovation, Level 6, 75 Talavera Road, North Ryde, 2109 NSW Australia

**Keywords:** Social network analysis, Longitudinal research, Collaboration, Brokers, Centrality, Consumer engagement, Implementation science

## Abstract

**Background:**

In 2012 and 2013, we conducted a social network survey of a new translational research network (TRN) designed to deliver better care to cancer patients. Results of these two surveys showed that silos of researchers and clinicians existed before the TRN was established but that the network had mediated collaborative relationships. This paper reports on a third social network survey of the TRN and focusses on the structure of the collaborative arrangements among members.

**Methods:**

Members of the TRN were invited to complete an on-line, whole network survey in May 2015. The survey asked respondents to identify personal impacts, outputs and wider outcomes attributable to their TRN membership. The final question asked respondents to select the name of TRN members with whom they had collaborated either formally or informally. For each member nominated, they were asked to say whether they had known this person before joining the TRN.

**Results:**

Response rate was 70 %. Over 4 years, the TRN has grown in size from 68 to 244 members. Relationships within and across the TRN have become more collaborative and interactive, with 1658 collaborative ties between members and over 40 % of ties with people unknown to participants before they joined the TRN. This points to a well-functioning network which has retained its focus on the original goals of the TRN and has fostered collaboration between researchers, clinicians, managers, consumers and TRN operational staff. This survey shows that the TRN’s impact goes beyond outcomes from formal TRN-funded projects. About one third of respondents could list projects not directly funded by the TRN but which are attributed to TRN membership. Examples of practice change brought about through the TRN were given by 77 % of respondents. A substantial risk factor for the future is the high levels of dependency on key or central TRN participants.

**Conclusions:**

The structure of the TRN with its active central actors and brokers has been able to foster collaboration on implementation initiatives that result in practice change. The role of a social professional network in driving this collaboration is shown.

**Electronic supplementary material:**

The online version of this article (doi:10.1186/s13012-016-0381-y) contains supplementary material, which is available to authorized users.

## Background

Translational research (TR) or more particularly, T2 practice-based research, aspires to take biomedical discoveries “from the laboratory bench” and transform them into useful clinical practice “at the bedside.” This endeavour benefits from collaboration of stakeholders with expertise and understanding from research, clinical and consumer viewpoints to work out ways to achieve this translation [1–3]. Translational research networks (TRNs) are an attempt to provide a supportive structure that can drive collaboration by providing access to new and diverse research partners, funding, research infrastructure, and project administrative support [4, 5]. As a means of providing an enabling environment in which translational research can be designed and carried out to bring about successful and sustained practice change, TRNs are an important implementation strategy. Increasing personal and collaborative links between clinicians and researchers breaks down the “us and them” mentality, based on discipline-specific paradigms. Interventions are known to fail if they do not pass individuals’ test of being acceptable, feasible, appropriate and credible [6]. Interventions co-designed by an interdisciplinary team are therefore more likely to be successful as all viewpoints and from different areas of expertise are considered and respected. As such, the study of the structure and function of TRNs is of key interest to those undertaking knowledge translation and implementation activities.

Social network analysis is a useful approach for studying TRNs and provides a valuable theoretical framework to consider information flows, social and professional influence and the phenomenon of siloed thinking and operating [7]. A social network is a group of people with ties or links between them [8]. It is the relationships between the members that are of primary interest rather than the characteristics of the people themselves [9]. Social network graphs, or sociograms, are built by surveying members and collecting self-reported information on the relationship in question. Each survey produces a snapshot of the network but can, by re-surveying at different points in time, provide useful longitudinal data. Parameters can be computed from these sociograms to define the structure of the network such as the density (how many actual ties there are compared to the possible number of ties), which members interact the most with other members (the central key player role) and who is in a go-between role linking otherwise unlinked members (the broker key player role). By identifying the individual members’ attributes (e.g. geographic location, profession), the influence of commonly reported silos on choice of collaborative partner can be examined.

### Work to date

In previous work [7, 10–12], we examined the establishment and growth of a TRN in the field of T2 cancer research from its foundation in 2011 over its 4 years of operation. The TRN was established after achieving funding as a result of a competitive grant process and was largely built around the core foundational members’ current and prior research contacts. Its 68 members were drawn from a range of hospitals, universities and research centres, and members were from disciplines as diverse as palliative care, protein chemistry, health economics and genetics. A governing body made up of 14 members was established to guide and oversee the network. A network director, manager and several network operations staff were appointed. The TRN was focused on T2 cancer research: introducing clinically proven knowledge of cancer processes, diagnostic or treatment regimes into routine clinical practice. The TRN also provided access to funding for 1-year projects, conference and professional development grants, shared databases and facilities and support from project officers and translational research fellows. Membership was open to researchers, clinicians and students of the member organisations who applied and was free. A consumer advisory group made up of people with cancer or who have survived cancer was convened to have input into research priorities and design.

This previous work [7, 10–12] demonstrated that before the start of the network, silos and tightly bound structures pervaded activities [11]. Relationships were based primarily on profession (clinicians or researchers) and geographic location. Using a social network survey (collaboration survey #1) in 2012, we were able to show that the presence of the TRN helped to mediate network relationships, appeared to close the profession gap between researchers and clinicians and influenced increased connectivity between partners. These analyses, supplemented by interviews [12], also highlighted how key players acted as go-betweens to link people and resources and that the network manager was the primary broker in this process [10]. Geographic location was associated with members’ choice of strategic partners, with network participants preferring to work with other local network participants [11]. At that early stage, it was not possible to identify and therefore to link this to any network outcomes. The collaboration survey #2, conducted in 2013 after 18 months of operation, was administered to a much larger network (268 members). The response rate for this second survey (43 %) was much lower than the first round (76 %) so the results of the social network component were viewed with caution. Over a quarter of the respondents in the sample (28 %) stated they had changed their practice (primarily around universal consenting for a tumour tissue bank and the inclusion of consumers in research design) and acknowledged personal benefits from TRN membership.

### Aims

This paper reports on the results of collaboration survey #3 in May 2015 after 4 years of network operation. Over this time, the TRN had lost and gained some members but the core of original members present at the foundation in 2011 had remained largely constant. Notably, there were now nine consumer advisors. Many of the questions were the same as the previous two surveys allowing a comparison with previous years.

This study has two objectives. We firstly aimed to evaluate the TRN’s performance over the 4 years of its operation in terms of translational research planning, projects, outputs and dissemination. Few papers providing longitudinal evaluative data of a TRN have been published. Secondly, the study informs TRNs generally or any other organisation seeking to drive collaboration by examining the structure of collaborative relationships and looking at key player roles.

The study examines collaboration between members but defines it as both formal and informal links. Collaboration has been defined as the interaction of two or more independent partners, each of whom bring a unique view or expertise to a common problem [13]. Collaboration is a key success factor in knowledge translation [3] and implementation [1, 2]. Studies that consider formal collaboration such as co-authorship on papers or funded research partnerships [14, 15] may miss the important hidden work of a socio-professional network [16] in the context of the complex adaptive systems of health [17] and research: the corridor conversations, the advice over the phone, the go-between’s role in securing resources or introducing to a key person. We designed the study to capture these informal relationships and used them, rather than just the formal ties of funded project partners, to assess TRN collaborative outcomes.

## Methods

Ethics approval was obtained from the appropriate local health district Human Ethics Committee (LNR/11/254). The survey was based on the first two collaboration surveys undertaken by the TRN and was piloted by 12 members and refined and reformatted in response to their comments.

People listed as members of the TRN, as of January 2015, were invited to complete an on-line, whole network survey in May 2015, with each member receiving a link to a secure survey site via personal email. Respondents were assured of anonymity in the reporting of results (names being replaced by anonymous codes) and were required to provide formal consent. The survey was a whole network survey [18, 19], that is, we sought answers from all members to reflect the whole network, rather than a sample of members. To maximise the response rate, three rounds of follow-up reminders were emailed to non-respondents over 4 weeks. Prior to the final reminders, the network manager requested research group leaders to encourage non-respondents in their groups to complete the survey. Table 1 summarises the survey administration.

**Table 1:** Summary of TRN survey #3 administration

Dates	Reminder	Number of emails sent	Surveys completed
12 May 2015	First email invitation	244	91
21 May 2015	First reminder email	176	33
27 May 2015	Second reminder email	142	32
28 May 2015	Email sent to research group leaders		
29 May 2015	Final email reminder	87	35
	Total		192

The survey was divided into two sections. A de-identified version is provided in Additional file 1. The first section collected demographic and descriptive information that was not able to be sourced from TRN documents. As noted in our previous work [7, 11], clinicians (largely hospital-based) and researchers (largely academic, university-based) [20] can be viewed as coming from different cultures as their paradigms, language and *modus operandi* are different, exacerbated by increasing complexity and specialisation in both arenas. All three network surveys therefore asked respondents to indicate which of the two dominant paradigms they aligned with most by choosing a “self-title” from clinician, researcher and clinician-researcher. Manager was added as an extra option in the second and third surveys. Consumers skipped this question and were automatically put into the consumer self-title group.

To understand the process of network growth in numbers, the survey asked respondents if they were invited or influenced to join the TRN by another member and, if so, to name the member. An important success factor in both collaboration and in networks as a whole is a clear shared understanding of its purpose. To test this, respondents were also asked to briefly outline the objectives of the TRN as they understood them. Answers were collated and frequency of key words/concepts were recorded and then compared with the stated objective of the TRN to assess respondents’ understanding.

Two questions asked respondents about personal impacts of the TRN and one about the wider outcomes of the network. A free text question asked for any examples of an observed or personal change in practice resulting from TRN activities. Answers were aggregated into categories using key words and frequencies of each were noted.

The last part of the survey asked about respondents’ involvement in TRN events and activities and previous TRN-funded projects and to nominate any non-TRN-funded research collaborations resulting from TRN membership. A final question asked respondents to select the name of any TRN member with whom they had collaborated either formally (e.g. on a funded project) or informally (have discussed aspects of research, supplied equipment, advice or expertise). For each member nominated, they were asked to say whether they had known this person before joining the TRN.

Network parameters were calculated for the whole network and for each member using UCInet v6 [21] social network analysis software. Network parameters were network size, density, number of ties, reciprocity of ties, and the centrality and brokerage potential (betweenness centrality) for each member. These terms are defined in Table 2. Network diagrams were generated using NetDraw [22].

**Table 2:** Social network parameters and their definitions

Term	Whole network or individual?	Definition
Node	Both	A node is a member of a network
Tie	Both	A tie represents a self-reported link between two nodes
Density	Whole network	The number of actual ties divided by the number of possible ties. Reported as a percentage
Reciprocity	Whole network	The extent to which ties between any two nodes is acknowledged by both. If there is full agreement across the network then reciprocity = 1.0; no agreement = 0.0
Clustering	Whole network	The extent to which nodes are grouped by an attribute
E-I Index	Whole network	External-internal index looks at actual ties versus expected ties relating to a certain attribute. It compares ties to members within that group (internal) to those outside (external) that group. Results are between −1.0 and +1.0 and have a *p* value
Degree	Both	Number of ties per node (either nominated by others or by the member themselves)
Indegree	Individuals	Number of ties reported by others directed to the focal member
Outdegree	Individuals	Number of ties reported by the focal member
Centrality	Individuals	Members with the highest interaction (ties to and from) with others
Betweenness centrality	Individuals	Members who have brokerage potential as they lie on the shortest path between two nodes that are not directly linked

Analysis was undertaken on the current survey then compared, where appropriate, with results from the previous two surveys. As the response rate was low for the second survey, caution was taken in these comparisons.

## Results

### Demographics

The response rate for the on-line survey was 79 % (192/244). Of these, 21 (9 %) respondents formally refused consent (question 1), leaving 171 completed surveys (70 %, 171/244) although some questions were skipped by some respondents. The average time to complete the survey was 14 min. Respondents from all subgroups of the TRN were represented. As in the first two surveys, worksites were divided into three groups (designated central, satellite and peripheral) for analysis, based on their geographic and administrative proximity [23]. Central sites had high proximity, satellite sites medium, and peripheral low. Sixty-three percent of respondents worked at a central site, 28 % at a satellite site and 9 % at a peripheral site. A comparison of respondents and non-respondents showed that they were similar in gender distribution (*χ* ^2^ (1, *n* = 244) 0.87, *p* = 0.35) but representation from central, satellite, peripheral sites were dissimilar (*χ* ^2^ (2, *n* = 235) 6.97, *p* = 0.03). Satellite sites were least well represented and peripheral sites the best. All but one consumer participated (8/9). Table 3 summarises these results.

**Table 3:** Comparison of respondents and non-respondents

	Respondents (%)	Non-respondents (%)
Gender		
Female	116 (68 %)	45 (61 %)
Male	55 (32 %)	28 (38 %)
Proximity		
Central	38 (22 %)	15 (21 %)
Satellite	11 (6 %)	13 (18 %)
Peripheral	114 (67 %)	44 (60 %)
(Consumers)	8 (5 %)	1 (1 %)

Nearly half of the respondents (80 or 47 %) chose “researcher” as their self-title, 22 (12 %) respondents chose “clinician”, 16 (9 %) chose “manager”, 18 (11 %) chose “clinician-researcher” and 20 (4 %) chose “other”. These “others” were aggregated into one of the four categories based on their “If other please specify” response plus their response to their main activity. For example, research nurses were aggregated with “clinician-researcher” and doctoral students to “researcher”. Five percent (8/171) of respondents were consumers.

### TRN objectives

There were 162 responses to the question: “Briefly, what is the main objective of the TRN as you understand it?” Answers matched fairly closely to the stated overall goal of the TRN, i.e. “to develop a sustainable translational research engine and to apply it to identified areas of need”. Frequently used words were research (126 times), collaboration/network (38), translation/al (42), facilitate/support (31), consumers/patients (17), grants/funding (7) and bridge/bring together (6). Notable was the absence of the concept of sustainability; it was only mentioned in two answers.

### TRN invitations

Sixty-nine respondents (40 %) nominated 77 members who had invited or influenced them to join the TRN. The network director was nominated the most (16 times), followed by two researchers five times each, and a large peripherally located research group was named four times.

### TRN personal impacts

Personal impacts of the TRN were assessed using a Likert scale of responses (from “not at all or not yet” to “to a large extent”). About two thirds of the respondents felt that TRN had, at least to some extent, increased their knowledge and skills, increased their career and networking opportunities, provided access to research opportunities, support and expertise and had been involved in TRN activities. The most positively viewed statements were *TRN has given me opportunities to meet and/or work with new people* (68 % scoring “To some extent”—“To a large extent”); *TRN has given me access to useful resources* (68 % scoring “To some extent”—“To a large extent”) and *The time I have invested in TRN has been useful* (70 % scoring “To some extent”—“To a large extent”). On this last statement, 18 % answered “To a large extent” The least positively viewed statement was *TRN has got me interested in taking part in research* where 25 % answered “Not at all/not yet”.

The most positively rated statement concerning the wider outcomes of TRN is *TRN has made it easier for people to find funding for projects, education and travel,* with 85 % scoring “To some extent” – “To a large extent,” with 21 % indicating “To a large extent”. Four out of five respondents also felt that, at least to some extent, the TRN had made it easier for people to get involved in translational research and to find collaborative partners and had increased the enthusiasm for translational research.

There were 122 responses to the free text question: *Can you give an example of a change in practice that has come about as a result of TRN activities?* Some respondents gave more than one example. Responses were thematically analysed and aggregated. Most nominated changes were engagement with consumers (42, 34 %), tumour tissue bank (35, 29 %), diagnostic improvements around hereditary breast, ovarian or colorectal cancer (5, 4 %), and improvements to pain assessment (3, 2 %). Other changes nominated by single respondents were: the role of the GP in continuing care of the cancer patient, use of Q-stream and Evi-Q, promotion of qualitative research design and changes in practice flowing on from a deeper understanding of patients’ issues as a result of involvement in research. There were 33 (27 %) respondents who wrote “not applicable”, “no” or “do not know” or explained they were not in a position to see any changes.

Consumer engagement was seen as having a number of benefits. Examples of responses are consumers provide valued input into early research design and planning for trials, they provide helpful feedback on design of consumer aids and meeting consumers adds passion to a project when one can “put a face” on one’s objective.

### Contacts with other TRN members

Eighty-two percent of respondents (133/162) had contact with other TRN members or staff. For the 29 (18 %) who had not had any contact, this was the final question in the survey. Contact with staff (77 %, 103/133) and attendance at a formal TRN meeting (75 %, 100/133) were the two most nominated activities.

Most respondents (77 %, 103/133) had not been involved in one of the TRN-funded projects over the last 4 years. Twenty-nine (22 %) respondents nominated other projects not funded by TRN that had resulted nonetheless through TRN membership. Table 4 summarises the projects nominated, illustrating their broad nature.

**Table 4:** Selection of (de-identified) answers asking for brief details of projects not funded by TRN but coming about as a result of TRN involvement

Responses	
Cancer diagnosis techniques	
Data and auditing research	
Molecular research on obesity and diabetes	
Lifestyle changes to reduce cancer risks	
Bio markers for cancers	
Telomere research	
Pain medication errors	
Projects with consumer agencies	
Health literacy issues	
Health economics aspects of cancer treatments	
Survivorship following germ cell tumour diagnosis	
Reporting of complications by outpatients undergoing cancer treatment	
Identifying trends in national cancer incidence and trends.	
Cancer and exercise studies	

### Collaboration network diagrams

**Fig. 1:**
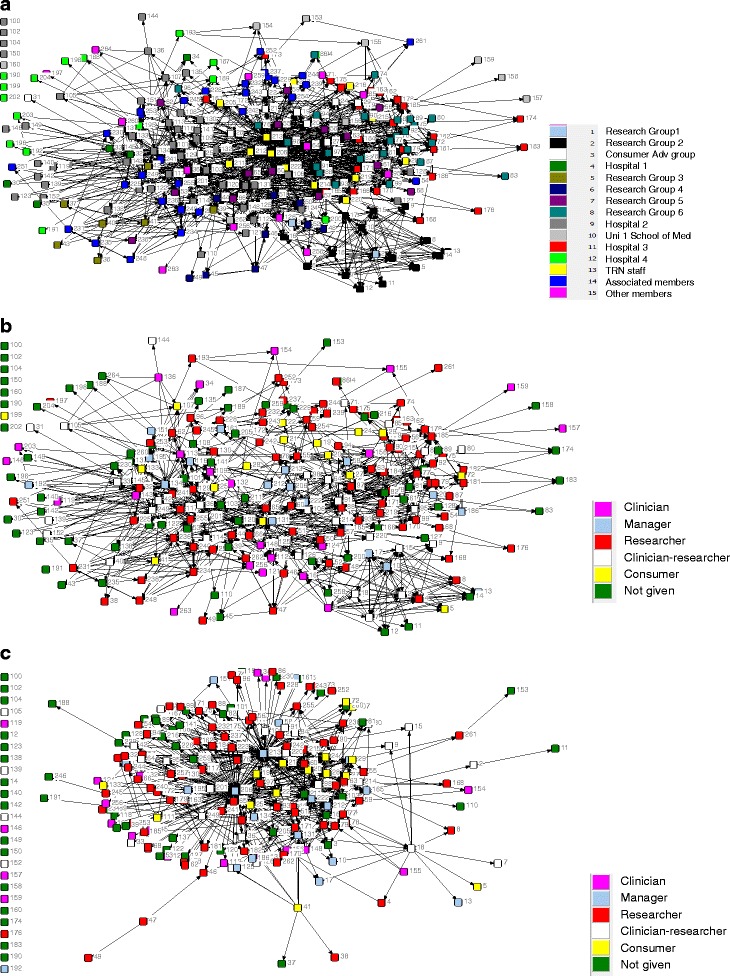
**a** Collaboration network. Please select those people with whom you are currently collaborating on a TRN activity, event or project. By “collaboration” we mean formally (e.g. on a funded project) or informally (e.g. have discussed aspects of research, supplied expertise, advice or equipment to others). **b** Collaboration network. Only showing ties of people who knew each other before the network started. Coloured by self-title. **c** Collaboration network: only showing ties of people who did not know each other before the network started. Coloured by self-title

Figure 1a–c show the collaboration network diagrams. Each point (node) represents a respondent, and each line (tie) represents a collaborative link. The structure of this network was analysed using UCInet [21]. The number of ties each respondent has (degree) and the number of times a respondent lies on the shortest path between two other respondents therefore being in a brokerage position (called betweenness centrality) were computed for each respondent.

Network size was 244, density was 3.9 % and there were 1658 ties. The average number of ties per node was 11, and the range was 0–168 ties. There were 987 ties between members who knew each other before the network started and 671 ties between people who did not know each other before the network started.

Respondents with the highest involvement with other respondents were the network manager (ID number, 206), a TRN staff member (213), a clinician-researcher (36) and the network director (131). For both outdegree and indegree (number of ties the member reports and the number of ties reported by others directed to them, respectively), 206 and 213 are the two most central actors. They were also the two people who were in the strongest brokerage positions, that is who were on the shortest path between two members who were not directly linked. Tables 5, 6 and 7 give numbers of ties and betweenness centrality measures.

**Table 5:** Members with the highest outdegree i.e. who have nominated the most collaborative ties with other members

ID	Name	No. of ties they report
206	Network manager	151
213	Network staff member	125
36	Clinician-researcher	50
134	Clinician manager	42
165	Researcher	42
131	Network director	41

**Table 6:** Members with the highest indegree, i.e. who have been nominated the most as collaborative ties by other members

ID	Role	No. of ties directed to them
206	Network manager	47
213	Network staff member	34
131	Clinician-researcher	31
36	Clinician-researcher	26
65	Researcher	23
222	Network staff member	22
81	Researcher	20
60	Researcher	19
97	Researcher	19
103	Clinician-researcher	19

**Table 7:** Members with the highest brokerage potential as members on the shortest path between two otherwise unlinked members

ID	Role	Betweenness or brokerage potential
206	Network manager	11162
213	Network staff member	5132
36	Clinician-researcher	2740
131	Clinician-researcher	2241

Reciprocity measures how often a tie is nominated by both parties. If we take out non-respondents who could not reciprocate (*n* = 171), reciprocity is 32 %, which is relatively low.

Members, on average, did not collaborate with a member of the same site group (E–I index, −0.88; *p* = 0.28) or with someone with the same self-title (E–I index = −0.88; *p* = 0.40) more than they did with members from outside these groups. In other words, there is no obvious pattern of clustering around sites or professions when averaged across the whole network.

### The nature of collaboration

The social network questions were deliberately placed at the end of the survey, after questions about consideration of personal impacts, wider outcomes and specific involvement in TRN events and activities. These diverse aspects of TRN involvement “primed” people to answer the final question using a broad socio-professional understanding of collaboration: “In the next and final question we want to know with whom you are collaborating. By “collaboration” we mean either formally (e.g. on a funded project) or informally (e.g. have discussed aspects of research, supplied expertise, advice or equipment to others) … Please select those people with whom you are currently collaborating on a TRN activity, event or project …” This allowed us to capture informal collaborative ties as well as the formal.

**Fig. 2:**
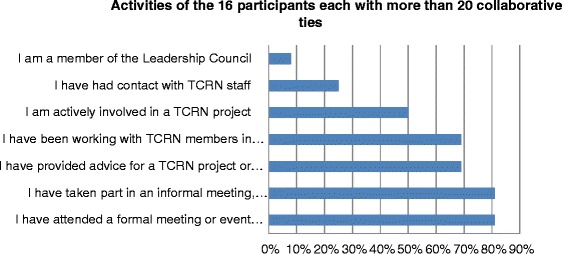
Involvement in TRN activities reported by the 16 participants who nominated the most collaborative ties (range 151–22)

**Fig. 3:**
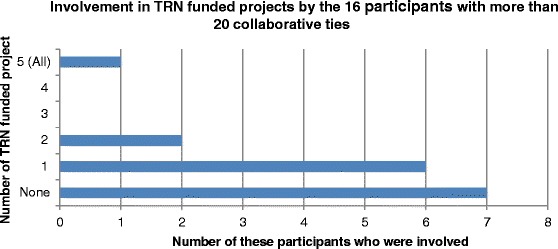
Involvement in TRN-funded projects reported by the 16 participants who nominated the most collaborative ties (range 151–22)

Responses of the 16 participants who reported the most collaborative ties (over 20 each) were examined to drill into the specific activities they had in mind. The majority (75 %, 12/16) reported involvement in five or more of the seven activities listed (Fig. 2). This analysis also showed that half (50 %, 8/16) were actively involved in a TRN project (confirmed by their answer in the next questions about TRN-funded project involvement (Fig. 3), and 81 % (13/16) had taken part in an informal meeting, discussion or email exchange about the TRN. This confirms that respondents saw both formally defined activities (such as investigator on a funded project) as well as informal activities such as email discussions or giving advice as collaboration.

### A longitudinal view

**Fig. 4:**
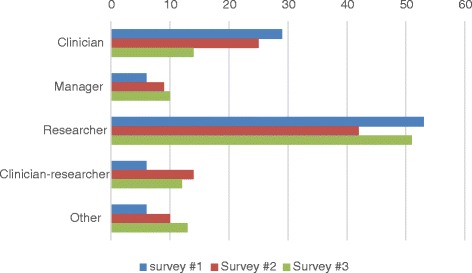
Percentages of self-title categories given by respondents across the three surveys

Detailed results from survey #1 have been reported elsewhere [10–12]. Table 8 summarises the main findings from the three collaboration surveys. Over this 4-year period, the network size grew from 68 members, to 263, then dropped back to 244. Figure 4 compares respondents’ self-title across the three surveys. A Pearson’s chi-square test (with *α* = 0.05) showed that there was no significant difference in the proportions of the different self-titles taking part: *χ* ^2^ (6, *N* = 271) = 10.72, *p* = 0.10. Density of the collaborative ties network in survey #1 (4 %) was the same as the density of collaborative ties in survey #3, but the size of these two networks was very different. For survey #1 only 26 members reported having a collaborative tie; there were 106 ties reported and only one tie was between two members who had not known each other before the TRN started. In contrast, collaborative ties in survey #3 involved 171 members and had 1658 ties, 671 of which were between people who did not know each other before the TRN started. The network manager appears in all three surveys as the central actor who interacts with the most other members. The manager is also the top broker in the network in both surveys #1 and #3.

**Table 8:** Comparison of the three collaboration surveys

	Survey #1	Survey #2	Survey #3
Date of survey	March 2012	April–May 2013	May–June 2015
Number of invitations to members	68	263	244
Response rate	76 % (2 % formally declined)	43 % (2 % formally declined)	79 % (9 % formally declined)
Density of collaboration network	4 % (pre-TRN = 31 %)	1 %	4 %
Number of respondents reporting their ties	26	94	171
Number of ties reported	106	326	1658
Number of new ties to people they did not know pre-network	1	119	671
Central actors’ ID numbers and title	131 (TRN director)	131 (TRN director)	206 (TRN manager)
	206 (TRN manager)	206 (TRN manager)	213 (TRN staff)
	262 (researcher)		
Brokers’ ID numbers and titles	206 (TRN manager)	131 (TRN director)	206 (TRN manager)
	165 (manager)	206 (TRN manager)	213 (TRN staff)
	81 (researcher)	(TRN staff)^a^	
	(Clinician)^a^	143 (clinician)	
	126 (clinician)		
	106 (clinician-researcher)		
Members nominated the most by new members as the person inviting or influencing them to join (ID numbers and titles)	NA	131(TRN director)	131(TRN director)
		44 (researcher)	165 (manager)
		236 (researcher)	44 (researcher)
		134 (manager)	Research group 1
Examples given of changes in practice as a result of TRN activities	NA	Answered by 28 % of respondents •Universal consent for the tumour tissue bank •Use of the pain modules •Involvement of consumers	Answered by 55 % of respondents •Universal consent for tumour tissue bank •Involvement of consumers •Diagnostic improvements around hereditary breast, ovarian or colorectal cancer •Improved assessment of pain

Due to its low response rate, survey #2 needs to be compared with caution

^a^No longer a member

Respondents of survey #1 were asked how they became a member. Half stated they were invited to join (50 %) and/or were involved in the original grant proposal for the network (47 %). Four percent joined on their own initiative while 10 % selected “other”. In survey #2, 38 (34 %) respondents stated they had been “involved from the start”, 44 respondents (44 %) stated they had been invited to join and 12 (11 %) said they joined on their own initiative. The network director and researcher 44 are key people in surveys #2 and #3 inviting others to join the TRN.

## Discussion

### Evaluation of the TRN

The survey provides evidence of a well-functioning network which has grown in size and complexity over the 4 years of its operation. Shared understanding of purpose is a key success factor in collaborative networks [24], and there is evidence that TRN members have this shared vision. The free text answers to the objectives of the TRN were closely aligned with the formally stated goal and emphasised research, translation, collaboration between researchers and clinicians, support, networking and consumer involvement.

There is also ample evidence of positive personal impacts of the TRN, wider network outcomes developing over time and a range of changes in practice. In addition to funded projects, TRN members are also collaborating on non-TRN-funded projects showing a positive influence on the wider research environment. Respondents reported attending formal events as well as being involved in less formal activities such as email exchanges about TRN issues.

Collaboration is widespread across the network both with existing and new contacts. Averaged across the whole network, clustering by site or within the self-title groups of clinician or researcher is not seen. A possible reason for this is the mixing effect of collaboration with consumers (who were coded as a separate site and self-title group) and the strong collaborative links with TRN Operational staff by many members of the network.

This pattern of widespread collaboration is evidence of a well-functioning network. For translational researchers, there is a clear need to collaborate. The very title “translational” points to moving from one domain to another or more graphically bridging the “valley of death” between bench and bedside [25]. Expertise from the research and clinical domains are both needed yet the increasing specialisation and “niche” nature of many research and clinical fields widens the valley [26, 27] and means that silos emerge more readily. Collaboration has long been hindered by these silos as they become isolated and introspective [28–30]. Language and concepts can be so specialised that it can be difficult to search effectively for a partner. Funding bodies expect these collaborations, not just across disciplines but to include consumers who can make a valuable contribution to research design.

Finding an appropriate collaborative partner is recognised as a barrier for practice-based TR involving more than just picking a name from a Google search or even a dedicated TRN website. Schleyer and colleagues [31] investigated the process of how biomedical researchers find potential interdisciplinary partners. They found that researchers highly prized compatibility in a potential collaborator in both personality and work style. This had a strongly social element in that they would more likely trust a personal recommendation from a colleague than a compatibility “hit” from a search engine on a website. The social structure of the TRN allows this level of facilitative social interaction. Another barrier is that disciplines can be so complex and specialised that researchers can often not know what they are looking for outside their own field. Again, the assistance of a boundary spanner in the TRN with some experience of both fields is invaluable [12]. The TRN members in key brokerage roles appear to be mediating these introductions in the TRN. Researchers may also suffer from power differentials in seeking a collaborator. Junior researchers or “less powerful” professions such as nursing or allied health with a limited social professional network can find it hard to find an appropriate partner, and it may be intimidating or awkward if a marked power differential exists [31]. Again, having go-betweens that can broker these collaborative introductions is a clear advantage in the TRN.

The number of new links between people who did not know each other before the start of the TRN has grown markedly since the first collaboration survey in 2012. At that time, only one respondent reported a collaborative tie with a person they had not known before involvement with the TRN. In this survey, 671 new ties are reported, giving clear evidence of the networking and collaborative opportunities TRN gives members. The TRN manager, director and staff have strong brokerage potential as do many of the governing body members. We know from previous work with the governing body [12] that their network position as a broker is enacted though giving advice, acting as a go-between or facilitator and sometimes as a “gate-jumper” (the opposite of a “gate-keeper”, i.e. achieving access to difficult to get resources or people). A strong case for the influence of the TRN on building this interdisciplinary collaboration can therefore be made.

Considering these 671 new collaborative ties, further, we examined whether these new ties were limited to TRN staff who may reasonably be expected to make new contacts as part of their job. By removing the network manager (206), the director (131) and operational staff (213) and (222), 283/671 ties (42 %) remained between respondents who did not know each other before the TRN started. This shows both that the TRN staff have been instrumental in building connectivity in the network and that other members have been able to find collaborative partners within the network through other means. The TRN website, for example, is frequently updated and features the work of members, providing a ready way to contact potential partners. Survey #3 showed members were involved in a range of TRN events and activities, which would also increase their chances of meeting a potential partner.

### TRN impacts and outcomes

This survey has been able to show that the TRN’s impact goes well beyond outcomes from formal TRN-funded projects. Only a small proportion of respondents were formally involved over the last 4 years with one of the TRN-funded projects (Table 9). This contrasts with our broad definition of collaboration that the majority of respondents reported (also reflected in differences between the members saying they were involved in TRN-funded projects and those officially listed (Table 9)). It also contrasts with the significant number of respondents reporting collaboration on other non-TRN-funded projects (Tables 5, 6 and 7). The diversity of these projects is large.

**Table 9:** Answers to “Over the last 4 years, were you involved in any of the TRN-funded projects?”

Project	No. who said they were involved (survey)	No. listed as investigators (TRN records)
2012 TRN-funded project	10	3
2013 TRN-funded project #1	3	2
2013 TRN-funded project #2	4	3
2014 TRN-funded project	11	4
2015 TRN-funded project	11	7
None of the above	103	225

There is evidence that most people attribute positive personal impacts and wider outcomes to their involvement with the TRN. The most positively received statement was “The time I have invested in the TRN has been useful” closely followed by the “The TRN has given me opportunities to meet and/or work with new people”. This latter statement is borne out by the number of new ties reported in the network. Translational research is focused on patient outcomes through changes in clinical practice so the long list of instances of changed practice that members attribute to the TRN is encouraging.

It is clear that consumer engagement facilitated by the TRN has had a significant impact. All nine consumers are people currently living with, or who have survived cancer, or people who have cared for a family member with cancer. They have all undergone research facilitation training. All formal grant proposals are presented to the consumer group for feedback. Comments on consumer engagement given in the survey included:

Consumer input to our project and having a cancer survivor speaking to the students was invaluable. When you put a face to the problem, it becomes personal and this made everyone in the lab more motivated to find a solution (Researcher, University 1)

My engagement with consumers has helped formulating my research proposal (Researcher, Laboratory Setting)

I believe researchers have a new understanding of the value of consumer representative involvement in their research - in all the contacts I have had with researchers in [the university] and other institutions (Consumer)

We have become more aware of the need to involve consumer advocates/representatives in our grant proposals. Hearing about how they think about issues of relevance has been an eye-opener. The TRN's consumer panel has been a useful avenue through which we were introduced to this (Researcher Research Group 4)

I have helped one researcher create forms for patients to become involved in trials, and will be helping another in the coming months (Consumer)

The tumour tissue bank was nominated frequently by clinicians as being a catalyst for change in their practice. Both the bank and consumer involvement were mentioned in survey #2. New to this current survey is work being done on diagnosis and management of hereditary cancers.

### Lessons for TRNs

This wealth of longitudinal data shows that silo-spanning, translational collaboration can be facilitated by strategic social interactions to result in positive personal impacts and wider outcomes. TRNs can become large and unfocussed if many new people join in an unstructured way. New members that join through the personal invitation of key members allow a manageable and strategic growth in numbers.

TRN staff such as the director, manager, operational staff and other key players can facilitate introductions of members from different areas to one another thereby solving a major barrier to strategic collaboration. Their knowledge of the wider research and clinical community allows them to target people to link up rather than a broader scattergun approach. These key players also have a significant role in facilitating access to resources. This has two implications for those managing TRNs: (1) people recruited to these roles should be good communicators and be active in making personal connections with members. (2) orientation to the organisations and familiarisation with the context in which they are working is important for them to have that overarching strategic role, and time should be invested in this. Once the introductions have been made, collaborations appear to flourish as seen by the number of non-TRN-funded partnerships members have initiated with other members.

A warning is also apposite here: the key players in the network need to share the role so that all the brokerage activity is not being done by a single actor. If this solo actor leaves the TRN, their absence is likely to disrupt the function of the network, fragment groups, or at the least slow the formation of new ties.

Consumer involvement is a powerful tool for designing high-quality, relevant studies as well as driving positive personal impacts and wider outcomes. Recommended is a formal process for recruiting and training appropriate consumer representatives to optimise their input into research design and practice. Again, these collaborative links between consumers and network members can be brokered by TRN staff.

### Strengths and weaknesses of the study

The study achieved a strong response rate (79 %) after careful preparation and concerted follow-up. The comparison of non-respondents with respondents showed that most sites were well represented. The lowest proportion of responses (6/18 members, 33 %) came from the hospitals 2 and 3. Groups with high response rates were research group 3 (8/9 members 89 %—ninth member formally opted out), the consumer advisory group (8/9 members, 89 %) and university 2 (23/33 members, 70 %).

Social network surveys collect self-reported ties. The reliability of this was maximised by using a roster style format, where names of all members were provided. This meant that respondents were not relying on recall alone. The list of 244 members had the potential to impose a huge respondent burden, but this did not seem to be a problem. The average time of completion was 14 min, and verbal feedback from the pilot verified that the roster of members, structured around research or clinical groups, was easy to work through.

While the formal measure of reciprocity of ties was low at 32 %, we can argue that this is not a significant issue in this context. The type of professional collaborative interaction we wanted to capture was deliberately broad, i.e. not just collaboration on formally funded projects but such things as casual conversations leading to loan of equipment, introductions to other key members and giving advice. The report of a tie by one party in this context could be argued to be indicative of an interaction of some sort which in this socio-professional context could justifiably be defined as collaboration. One striking example of this non-reciprocity is in the indegree and outdegree data of the most central actors. The network manager and TRN staff member 213 have nominated three to four times the number of ties to other members than ties others have directed to them (see Tables 5 and 6). This means that many people are forgetting or not defining as collaboration, contact with the network manager and staff. As TRN staff, they may be in a better position to accurately remember their interactions with other members.

This study examined a single translational network, but longitudinal data over 4 years shows a rich picture of how to foster cross-silo collaboration more generally. Additional file 2 discusses the methodological issues of adequate whole network response rates and respondent concerns over confidentiality.

## Conclusions

The TRN collaboration survey #3 has provided evidence of an active network that has grown in size and impact over the last 4 years of its operation. While relatively few respondents had been involved in TRN-funded projects, they reported 1658 collaborative ties with other members. Over 40 % of these ties were with people unknown to them before they joined the TRN. The network has retained the key central actors and brokers network manager, network director and clinician-researcher 36. TRN staff member 13 is now also a key player. Along with them, researcher 44 is a key nominator of new members. Insight from previous research suggests that members in brokerage positions in the network enact that role by facilitating collaborative links. Network outcomes such as changes in practice around consumer engagement and universal consenting for the tumour tissue bank are widely acknowledged by respondents and new diagnostic and treatment regimes for hereditary cancers are now also influencing practice. There seems little doubt that collaborative effort and stronger relationships have resulted since the establishment of the TRN.

The future of the TRN’s collaborative efforts and effectiveness rests on this platform of networked behaviours that is represented here. This future not guaranteed. It will have to be worked on, particularly in the transition to the next-round TRN, which is highly dependent on success in securing 5-year funding and ongoing collaborative efforts of key brokers and central actors.

## Additional files

Additional file 1: **De-identified version of collaboration survey #3.** Formatting of roster style social network questions shown (Q.18 ff). (18.1 kb) 

Additional file 2: **Some methodological considerations.**  Discussion of response rate, reciprocity and the high "opt out" rate. (14.4 kb)

## Abbreviations

TR: translational research
TRN: translational research network
