# Supplementary material for: Structuring successful collaboration: a longitudinal social network analysis of a translational research network
Source: Implement Sci. 2016 Feb 11;11:19. doi: 10.1186/s13012-016-0381-y (PMC4750242; doi:10.1186/s13012-016-0381-y)
Supplement: Supplementary file 1 — De-identified version of collaboration survey #3. Formatting of roster style social network questions shown (Q.18 ff). (18.1 kb) [file 13012_2016_381_MOESM1_ESM.docx]

**Additional File 1**

TRN Collaboration Survey#3

Welcome.

Thank you for taking part in the third TRN Collaboration Survey. The survey will help us to understand how the network has grown in the last year, our current patterns of collaboration and our progress so far. This survey includes a social network survey. These map the relationships between participants by surveying all members and then combining their answers. We need to use members’ names on the survey in order to construct each person’s web of contacts, however once submitted, data will be coded and de-identified. This is the same method we used for the first two TRN Collaboration Surveys.

**Individuals and institutions will be anonymous in all wider reporting of results**

This research has received Ethics approval from XX HREC [Approval number]. If you have any questions, please contact us [email].

The survey will take around 10 minutes to complete.

Please complete this survey by [date].

Q1 I have read the above information and agree to participate in this survey.

- Yes
- No thanks

If “No thanks” is selected then skip to end of survey

Q2 Firstly, what is your name?

__________________________

Q3 Are you a consumer representative on the TRN?

- Yes
- No

If “Yes” is selected, then skip to: Q7.

Q4 Which of the following makes up the major component of your work? (please select ONE)

- Direct patient care
- Administration or management
- Research
- Teaching / supervision of students or junior staff
- Other (please specify) ____________________

Q5 Which of the following titles would most closely describe you? (Please select ONE)

- Clinician
- Manager
- Researcher
- Clinician-researcher
- Other (please specify) ___________________

Q6 Briefly, what is the main objective of the TRN as you understand it?

_______________________________________________________

Q7 If you were invited or influenced to join the TRN by another member/s, please name them here.

_______________________________________________________

Q8. Please rate the following statements. As result of the TRN I have...

|  | Not at all or not yet |  | To some extent |  | To a large extent |
| --- | --- | --- | --- | --- | --- |
| Increased my knowledge and skills |  |  |  |  |  |
| Increased my career and networking opportunities |  |  |  |  |  |
| Been able to contribute my expertise and/or experience |  |  |  |  |  |
| Had access to research opportunities, support and expertise |  |  |  |  |  |
| Been involved in TRN activities |  |  |  |  |  |

Q9 Thinking about other personal impacts of the TRN so far, please rate the following statements.

|  | Not at all or not yet |  | To some extent |  | To a large extent |
| --- | --- | --- | --- | --- | --- |
| TRN has made me more aware of issues for patients and / or researchers |  |  |  |  |  |
| TRN has got me interested in taking part in research |  |  |  |  |  |
| TRN has given me opportunities to meet and / or work with new people |  |  |  |  |  |
| TRN has given me access to useful resources |  |  |  |  |  |
| The time I have invested in TRN has been useful |  |  |  |  |  |

Q10 Considering the wider outcomes of the TRN so far, please rate the following sentences. In my opinion, the TRN has:

|  | Not at all or not yet |  | To some extent |  | To a large extent |
| --- | --- | --- | --- | --- | --- |
| Made it easier for people to get involved in translational research |  |  |  |  |  |
| Made it easier for people to find collaborative partners |  |  |  |  |  |
| Made it easier for people to find funding for projects, education or travel |  |  |  |  |  |
| Increased the enthusiasm for translational research |  |  |  |  |  |
| Changed clinical practice |  |  |  |  |  |
| Changed research assumptions |  |  |  |  |  |

Q11 Can you give any examples of changed clinical or research practice that have resulted from TRN activities? E.g. consenting patients for the tissue bank; involving consumers in research design.

_______________________________________________________

Q12 Have you had any contact with other TRN members or staff so far?

- No (That is the end of the survey, thank you.)
- Yes

If no is selected, then skip to end of survey

Q13 What activities have you participated in with other members of the TRN so far? (Please select ALL that apply)

- I have attended a formal meeting or event run by the TRN
- I have taken part in an informal meeting, discussion or email exchange about the TRN
- I am actively involved in a TRN project
- I have provided advice for a TRN project or activity being run by others
- I have been working with TRN members in another way
- I have had contact with TRN staff
- I am a member of the Leadership Council

Q14 Over the last four years, were you involved in any of the TRN funded Projects? (Please select ALL that apply)

- 2012 Project A
- 2013 Project B
- 2013 Project C
- 2014 Project D
- 2015 Project E
- None of the above

Q15 Over the last four years, have you been involved in any new collaborations with TRN members that have resulted in new projects (that were not directly funded by the TRN)?

- No
- Yes (Please provide summary details.) ____________________

Q16 In the next and final question we want to know with whom you are collaborating. By “collaboration” we mean formally (e.g. on a funded project) or informally (e.g. have discussed aspects of research, supplied expertise, advice or equipment to others).

Please scroll down the list of names of all the TRN members.

Please select those people with whom you are currently collaborating on a TRN activity, event or project by clicking the first column.  For those you select, please also indicate if you knew the person before you joined the TRN.

Q18 TRN Members from Research Group A?

- Person 1 (Position e.g. Research fellow)
- Person 2 (Position e.g. Nurse Manager)
- I am not collaborating with anyone from Research Group A

If “I am not collaborating with...” is selected, then skip to Q20

Q19 Did you know these people before you joined the TRN?

Yes / No

Q20 TRN Members from Consumer Advisory Group

- Person 3
- Person 4
- I am not collaborating with anyone from Consumer Advisory Group

If “I am not collaborating with...” is selected, then skip to Q22)

Q21 Did you know these people before you joined the TRN?

Yes / No

Repeat for Person 5- 244

That is the end of the survey. We thank you for your time.
