# Supplementary material for: Structuring successful collaboration: a longitudinal social network analysis of a translational research network
Source: Implement Sci. 2016 Feb 11;11:19. doi: 10.1186/s13012-016-0381-y (PMC4750242; doi:10.1186/s13012-016-0381-y)
Supplement: Supplementary file 2 — Some methodological considerations. Discussion of response rate, reciprocity and the high "opt out" rate. (14.4 kb) [file 13012_2016_381_MOESM2_ESM.docx]

**Additional File 2**

**Some methodological considerations**

As with the first two surveys persuading members to respond to the survey was difficult. After the inadequate response rate of 42% in Survey #2 additional tactics were employed. Firstly the survey questions were pared down to lessen the respondent burden. Secondly, the membership list was carefully checked for currency and that all listed members had formally agreed to take part in TRN activities. This was highlighted as a shortcoming of the second survey; i.e. that some members thought they were “just on the TRN mailing list” and so were not required or eligible to take part. Thirdly, we asked that people who chose not to take part to log onto the survey and answer the initial question (“I agree to take part in this survey”) as no, thus opting out of future reminders. This resulted in 21 people formally declining. This was disappointing, but useful information for future surveys. Finally, the non-respondent list was supplied to the TRN manager after the second round of reminders had been sent out, allowing her to send a targeted personal email to Responsible Members encouraging them to support the survey with their non-respondent members. This resulted in a further 35 surveys completed. While these strategies have assisted in getting an adequate response rate, caution needs to be taken in interpreting the network data as the premise it is based on is that the whole network is mapped (rather than a sample).

The comparison of non-respondents with respondents showed that most sites were well represented. The lowest proportion of responses (6/18 members, 33%) came from the Hospitals 2 and 3. Groups with high response rates were Research Group 3 (8/9 members, 89% - ninth member formally opted out), the Consumer Advisory Group (8/9 members, 89%) and University 2 (23/33 members, 70%).

There is no definitive answer on what constitutes an “adequate” response rate for a whole network survey. Borgatti [[32](#_ENREF_32)] states there are two main considerations: the type of analysis being performed on the data (as some measures are more robust in the face of missing data than others [[33](#_ENREF_33)]), and the suspected structure of the network. If, for example the Network Manager (206) had been a non-respondent, the network structure would be a very different representation of the TRN. Since missing parts of the network are partly filled by respondents nominating non-respondents we can see that it is unlikely we have missed any key people.

Over 10% of respondents did not give consent and formally opted out of the survey. Possible explanations are lack of time, lack of interest, or concern over confidentiality issues. This has arisen in other social network surveys that we have administered, in spite of assurances to potential respondents and demonstrations of Ethics Committee Approvals and de-identification of data before presentation outside the TRN. Having formally opted out makes our follow up of these members inappropriate but would be useful information to have before launching another survey.

While the formal measure of reciprocity of ties was low at 32%, we can argue that this is not a significant issue in this context. The type of professional collaborative interaction we wanted to capture was deliberately broad: i.e. not just collaboration on formally funded projects but such things as casual conversations leading to loan of equipment, introductions to other key members, and giving advice. The report of a tie by one party in this context could be argued to be indicative of an interaction of some sort which in this socio-professional context could justifiably be defined as collaboration. One striking example of this non-reciprocity is in the indegree and outdegree data of the most central actors. The Network Manager and TRN staff member 213 have nominated three to four times the number of ties to other members than ties others have directed to them (see Table 6(a) and (b)). This means that many people are forgetting or not defining as collaboration, contact with the Network Manager and staff. As TRN staff they may be in a better position to accurately remember their interactions with other members.
